# Antiphospholipid IgG Certified Reference Material ERM^®^-DA477/IFCC: a tool for aPL harmonization?

**DOI:** 10.1515/cclm-2025-0032

**Published:** 2025-03-21

**Authors:** Claudia Grossi, Liesbet Deprez, Caterina Bodio, Maria Orietta Borghi, Suresh Kumar, Nicola Pozzi, Paolo Macor, Silvia Piantoni, Angela Tincani, Massimo Radin, Savino Sciascia, Gustavo Martos, Evanthia Monogioudi, Ingrid Zegers, Joanna Sheldon, Rohan Willis, Pier Luigi Meroni

**Affiliations:** Immunorheumatology Research Laboratory, 9354 IRCCS Istituto Auxologico Italiano, Cusano Milanino, Italy; European Commission, Joint Research Centre, Geel, Belgium; Dipartimento di Scienze Cliniche e di Comunità, Dipartimento di Eccellenza 2023-2027, University of Milan, Milan, Italy; Edward A. Doisy Department of Biochemistry and Molecular Biology, Saint Louis University School of Medicine, St. Louis, MO, USA; Department of Life Sciences, University of Trieste, Trieste, Italy; Rheumatology and Clinical Immunology Unit, ASST Spedali Civili, Department of Clinical and Experimental Sciences, University of Brescia, ERN-Reconnect Member, Brescia, Italy; Department of Clinical and Biological Sciences, University Center of Excellence on Nephrologic, Rheumatologic and Rare Diseases (ERK-Net, ERN-Reconnect and RITA-ERN Member) with Nephrology and Dialysis Unit and Center of Immuno-Rheumatology and Rare Diseases (CMID), Coordinating Center of the Interregional Network for Rare Diseases of Piedmont and Aosta Valley, San Giovanni Bosco Hub Hospital, University of Turin, Turin, Italy; International Bureau of Weights and Measures, Sèvers, France; Health and Digital Executive Agency of the European Commission, Brussels, Belgium; European Commission, Scientific Advice Mechanism, Brussels, Belgium; Protein Reference Unit, St. George’s Hospital, London, UK; Antiphospholipid Standardization Laboratory, University of Texas Medical Branch, Galveston, TX, USA

**Keywords:** antiphospholipid syndrome, anti-β2GPI, anti-cardiolipin, aPL-heterogeneity, Certified Reference Material (CRM), method-calibration

## Abstract

**Objectives:**

The Certified Reference Material (CRM) ERM^®^-DA477/IFCC is a new polyclonal IgG anti-beta2-glycoprotein I (anti-β2GPI) material for the harmonization of the laboratory diagnosis of antiphospholipid syndrome (APS). We evaluated CRM’s ability to represent the heterogeneity of APS patient anti-β2GPI antibodies and to calibrate IgG anti-β2GPI methods.

**Methods:**

We characterized CRM for its reactivity against domain-1, using the QUANTA Flash^®^ β2GPI-domain-1 assay, and against domains-4-5 of β2GPI, and single-domain-deleted β2GPI molecules using in-house ELISAs. We used QUANTA Lite^®^ ELISA, QUANTA Flash^®^ CLIA, and EliA™ FEIA methods to evaluate the CRM’s anti-Cardiolipin (anti-CL) activity. Four anti-β2GPI IgG methods (in-house and QUANTA Lite^®^ ELISA, QUANTA Flash^®^ CLIA, and EliA™ FEIA) were also used to evaluate the CRM’s calibration efficacy, alongside 133 clinical samples (CSs) and 99 controls.

**Results:**

The CRM showed high anti-domain-1 activity and no anti-domain-4-5 activity at the recommended assay dilution. The domain-dependent-β2GPI reactivity profiles were comparable with full-blown APS. There was acceptable dilution linearity for anti-CL assays with R^2^ ranging from 0.957 to 0.997. For the four anti-β2GPI IgG assays, calibration with the CRM led to a good comparability of the average result of CSs for two of the assays. New cut-offs calculated from this work improved comparability in quantitative results between three of the assays: 85 % concordance with CRM compared to 66 % concordance with assay-specific-calibration.

**Conclusions:**

The CRM is representative of patient anti-β2GPI/CL heterogeneity and should improve anti-β2GPI IgG method harmonization. However, the level of achievable method harmonization is affected by differences in the selectivity among the assays.

## Introduction

The goal of method standardization in laboratory medicine is to ensure that various measurement procedures targeting the same measurand will provide equivalent results, within an uncertainty consistent with medical decision requirements for any clinical sample (CS). The high number of variables in autoantibody measurements makes method standardization seem to be an unattainable goal. Patient autoantibody heterogeneity, technical differences among detection assays, and lack of common units to express results are the main pitfalls towards standardization [1], [2], [3], [4], [5], [6].

Laboratory testing for the autoantibodies associated with anti-phospholipid syndrome (APS) is no exception. APS is a multisystemic autoimmune disorder characterized historically by recurrent thrombosis and/or pregnancy loss and more recently by microvascular, hematologic and cardiovascular disease alongside a panel of biomarkers. Anti-phospholipid antibodies (aPL): anti-cardiolipin (anti-CL), anti-beta2 glycoprotein I (anti-β2GPI), and Lupus anticoagulant (LA) [7], 8] are part of the diagnostic criteria for APS but they also have a role in the disease pathogenesis. The key autoantigen of the syndrome is the five-domain blood glycoprotein β2GPI [7], 9], 10]. Antibodies directed against the Domain 1 of β2GPI (anti-D1) have a recognized pathogenic role in APS patients [11]. Interestingly, a few patients show a reactivity against D4 and 5 of the target-protein (anti-D4-5). These antibodies are considered non or less pathogenic, mainly present in aPL-positive subjects without clinical events (aPL carriers), and they fail to induce thrombi in rats [6], [12], [13], [14]. Including reactivity with D4 and 5 may improve APS profiling.

The development of stable, homogeneous and commutable reference materials can help laboratory testing meet aPL heterogeneity. The ideal reference material would be a polyclonal immunoglobulin preparation representative of the autoantibody subpopulations spontaneously occurring in patients: the use of pooled patient serum samples is a good strategy for this. In contrast, affinity purified materials used as polyclonal reference standards or monoclonal materials have some pitfalls. Affinity purified materials might offer only some autoantibody subpopulations, for example missing those with low avidity, and their long-term production/stability might be difficult. Monoclonal materials are easier to reproduce without significant differences among batches. However, both xenogenic and human monoclonal material from patients’ B cells are suffering from the limitation due to the reactivity against a single epitope. They are usually high-avidity immunoglobulins against linear epitopes, which contrasts with low-avidity and reactivity against conformational epitopes displayed by natural autoantibodies [1], 5], 6], [15], [16], [17], [18]. Improvements in method harmonization should reduce the variability in the diagnosis and classification of APS [8], 19]. Currently, there are four major methodological formats associated with these analytes: manual or semi-automated ELISA, and automated systems based on chemiluminescence (CLIA), fluorescence (FEIA), and Luminex multiplex immunoassays. The results generated by the ELISA methods were typically those used in establishing clinical cut-off and positivity-range values; however, those values show little comparability to those generated by the increasingly common (automated) CLIA, FEIA, and Luminex multiplex methods. Even though the new laboratory classification criteria require the use of ELISA for the inclusion in the clinical trials only, it is clear the need for standardization/harmonization of the quantitative results and, where appropriate, the qualitative results for the diagnostic setting [19].

A Certified Reference Material (CRM) ERM^®^-DA477/IFCC from the EC-JRC (listed in the JCLTM database) and the measurement standard 21/266 from NIBSC (established as the first WHO international standard) for anti-β2GPI IgG are now available [1], 20], 21]. These reference materials are identical as they were produced as one batch, split into two after production [22]. This polyclonal IgG anti-β2GPI material has good potential to represent APS patient anti-β2GPI IgG heterogeneity [1], 20], 21], being a pooled patient serum sample standard with an assigned arbitrary value in International Units (IU). The commutability of the CRM was evaluated by comparing its measurement results obtained with five manual ELISA and one automated CLIA platform with the measurement results obtained for routine clinical samples [21]. The results suggested that CRM is commutable. However, this study had several limitations, with a limited number of clinical samples giving reliable measurement results for each assay, and the increasingly used automated FEIA platform was not included [21].

In this manuscript, we describe the outcome of several additional studies performed to evaluate whether the CRM represents the heterogeneity of anti-β2GPI IgG in patients and whether improved harmonization of results could be achieved when assays measuring anti-β2GPI IgG were calibrated with CRM ERM^®^-DA477, and results are expressed in IU/mL. The CRM was characterized using D1 and D4-5 fragments and tested against a library of five novel recombinant β2GPI variants in which each of the five domains was systematically removed one at a time while retaining the other four. Since β2GPI binds cardiolipin [9], 23] and, therefore, anti-CL assays often identify anti-β2GPI antibodies rather than antibodies directly binding to CL [7], 24], 25], we also tested CRM in three commercially available anti-CL IgG kits.

## Materials and methods

### CRM preparation

CRM was prepared according to the manufacturer’s specifications. Further information is available in the supplementary file (Supplementary Figure S1).

### Characterization of CRM aPL profile

#### CRM’s anti-D1 and anti-D4-5 activity

Reactivity of CRM toward D1 fragment was evaluated using the commercially available QUANTA Flash^®^ B2GP1-Domain 1 (701188) assay (Inova Diagnostics, San Diego, CA, USA). Reactivity of CRM toward D4-5 fragment was evaluated using an in-house ELISA assay, as described earlier [12]. Further information can be found in the supplementary file. All measurements were conducted in triplicate.

#### CRM’s activity against specific domain deleted β2GPI molecules

**Figure 1:**
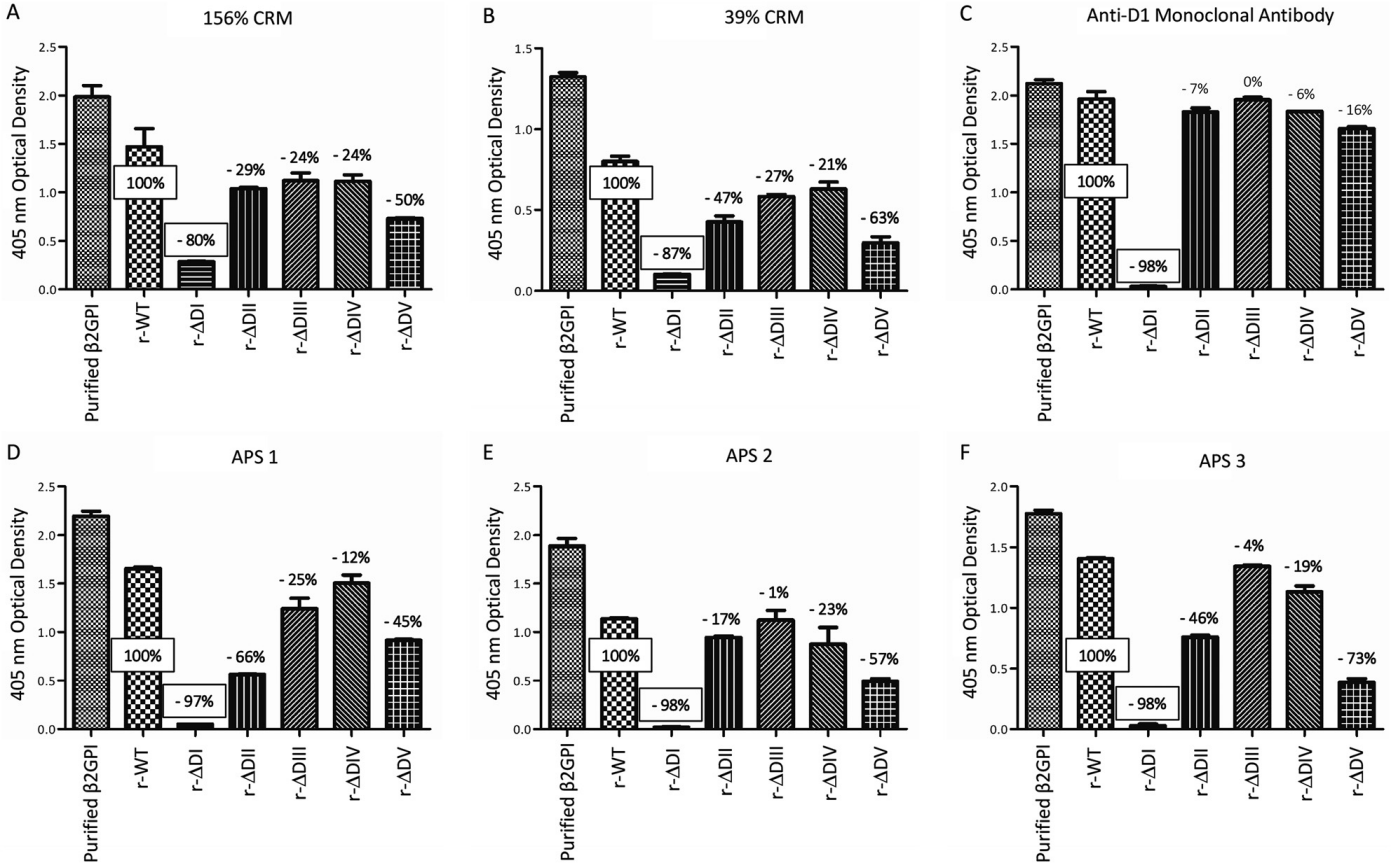
CRM’s, MBB2’s and three APS serum samples’ activity against β2GPI purified from human serum, recombinant β2GPI wild-type and a library of 5 novel recombinant β2GPI variants in which each of the five domains was systematically removed one at a time while retaining the other four. Reduction activity percentages relative to recombinant β2GPI wild-type are reported. (A-B) CRM tested at 156 % (1:32 dilution) percent dilution relative to the dilution factor recommended to test serum samples in the assay 1:50=100 %, and 39 % (1:128). (C) Anti-D1 monoclonal (IgG) antibody MBB2 tested at 2.5 μg/ml. (D-F) Full-blown APS serum samples evaluated at the recommended assay dilution (1:50, 100 %). APS 1 (thrombotic) and APS 2 (obstetric) are two triple (anti-β2GPI, anti-CL, and LA) positive APS patients. APS 3 (thrombotic and obstetric) is a double positive (anti-β2GPI, anti-CL) APS patient. r-WT=recombinant β2GPI wild-type; r-ΔDI=variant lacking domain 1; r-ΔDII = variant lacking domain 2; r-ΔDIII=variant lacking domain 3; r-ΔDIV=variant lacking domain 4; r-ΔDV=variant lacking domain 5.

Human β2GPI was purified from serum pooled from 100 healthy donors, as described earlier [14], 26]. Recombinant β2GPI wild-type (r-WT) and variants lacking domain 1 (r-ΔDI), domain 2 (r-ΔDII), domain-3 (r-ΔDIII), domain-4 (r-ΔDIV) and domain V (r-ΔDV) were produced in HEK293 cells by Dr. Kumar in the group of Pozzi using methodologies described recently [10], [27], [28], [29]. Purity was >98 %, as judged by SDS-PAGE. Serum-purified β2GPI and recombinantly made β2GPI WT and variants were used in ELISA assays, as specified in the supplementary materials. The reactivity of CRM was compared with three full-blown APS samples as well as with the monoclonal antibody MBB2, which binds to a conformational epitope in Domain I [28], 30]. More details are reported in Figure 1 legend.

#### CRM’s anti-cardiolipin activity

**Figure 2:**
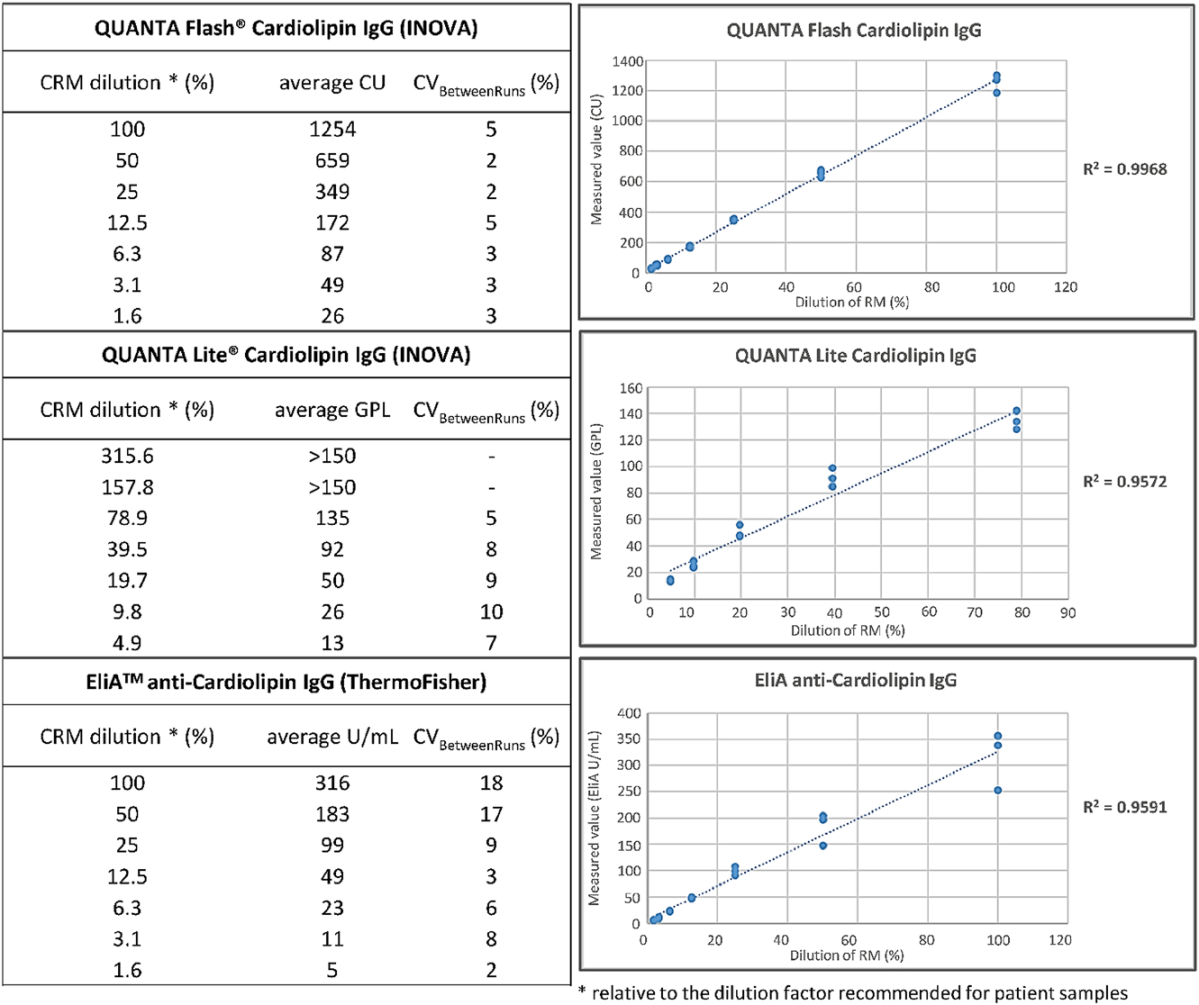
Dose-dependent anti-cardiolipin IgG reactivity of the CRM determined with three commercial assays. The dilution factor recommended to test patient samples corresponds to the 100 % CRM dilution, i.e. undiluted for QUANTA Flash^®^ aCL IgG and EliA™ cardiolipin IgG – not taking the applied instrument dilution factor into account -, and 1:101 for QUANTA Lite^®^ ACA IgG. According to this, we tested CRM by QUANTA Flash^®^ aCL IgG (701230) and EliA™ cardiolipin IgG at the following test conditions: 100 % (undiluted), 50 % (1:2), 25 % (1:4), 12.5 % (1:8), 6.3 % (1:16), 3.1 % (1:32), 1.6 % (1:64), corresponding dilution excludes the applied instrument dilution factor; and by QUANTA Lite^®^ ACA IgG III (708625) at the following: 315.6 % (1:32), 157.8 % (1:64), 78.9 % (1:128), 39.4 % (1:256), 19.7 % (1:512), 9.8 % (1:1,024), 4.9 % (1:2,048).

Reactivity of CRM toward cardiolipin was assessed using three commercial assays: QUANTA Flash^®^ aCL IgG (701230), QUANTA Lite^®^ ACA IgG III (708625) (Inova Diagnostics, San Diego, CA, USA), and EliA™ Cardiolipin IgG (Thermo Fisher Scientific, Freiburg, Germany). All measurements were conducted in duplicate, on three independent runs. Details are reported in Figure 2 legend.

### Clinical samples

We collected 133 aPL-positive serum samples and 99 age and sex-matched control samples. The aPL-positive cohort consisted of samples that were found positive for anti-β2GPI IgG/IgM and/or anti-Cardiolipin IgG/IgM and/or LA. Clinical characteristics are summarized in Table 1.

**Table 1:** Clinical and demographic characteristics of the aPL positive serum samples and control samples included in the commutability study.

aPL Positive serum samples (n=133)				
Diagnosis	aPL Carrier (n=34)	Thrombotic (n=67)	Obstetrical (n=14)	Thrombotic and obstetrical (n=18)
PAPS 64/133 (48 %)	/	39/67 (58 %)	13/14 (93 %)	12/18 (67 %)
SAPS 35/133 (26 %)	/	28/67 (42 %)	1/14 (7 %)	6/18 (33 %)
aPL carrier with SARDs 18/133 (14 %)	18/34 (53 %)	/	/	/
aPL carrier without SARDs 16/133 (12 %)	16/34 (47 %)	/	/	/
Demographic features	Age (average ± SD)		Females	
	48 ± 11		104/133 (78 %)	

**Control serum samples (n=99)**				

Diagnosis	Age (average ± SD)		Females	
aPL-negative SLE 24/99 (24 %)	43 ± 16		21/24 (87 %)	
Healthy donors 75/99 (75 %)	49 ± 9		56/75 (74 %)	

aPL, anti-phospholipid antibodies; PAPS, primary anti-phospholipid syndrome; SAPS, secondary anti-phospholipid syndrome; SARDs, systemic autoimmune rheumatic diseases; SLE, systemic lupus erythematosus.

Serum samples that were otherwise destined for disposal were collected from the routine diagnostic lab over the past 10 years. These samples were pseudonymized and stored at −20 °C. The samples were then thawed, and assay-specific aliquots were prepared and immediately refrozen at −20 °C. Any turbid, icteric, or hemolyzed samples were excluded from the process.

The Ethical Committee of Istituto Auxologico Italiano approved the study (22072010).

### Anti-β2GPI IgG measurements

**Figure 3:**
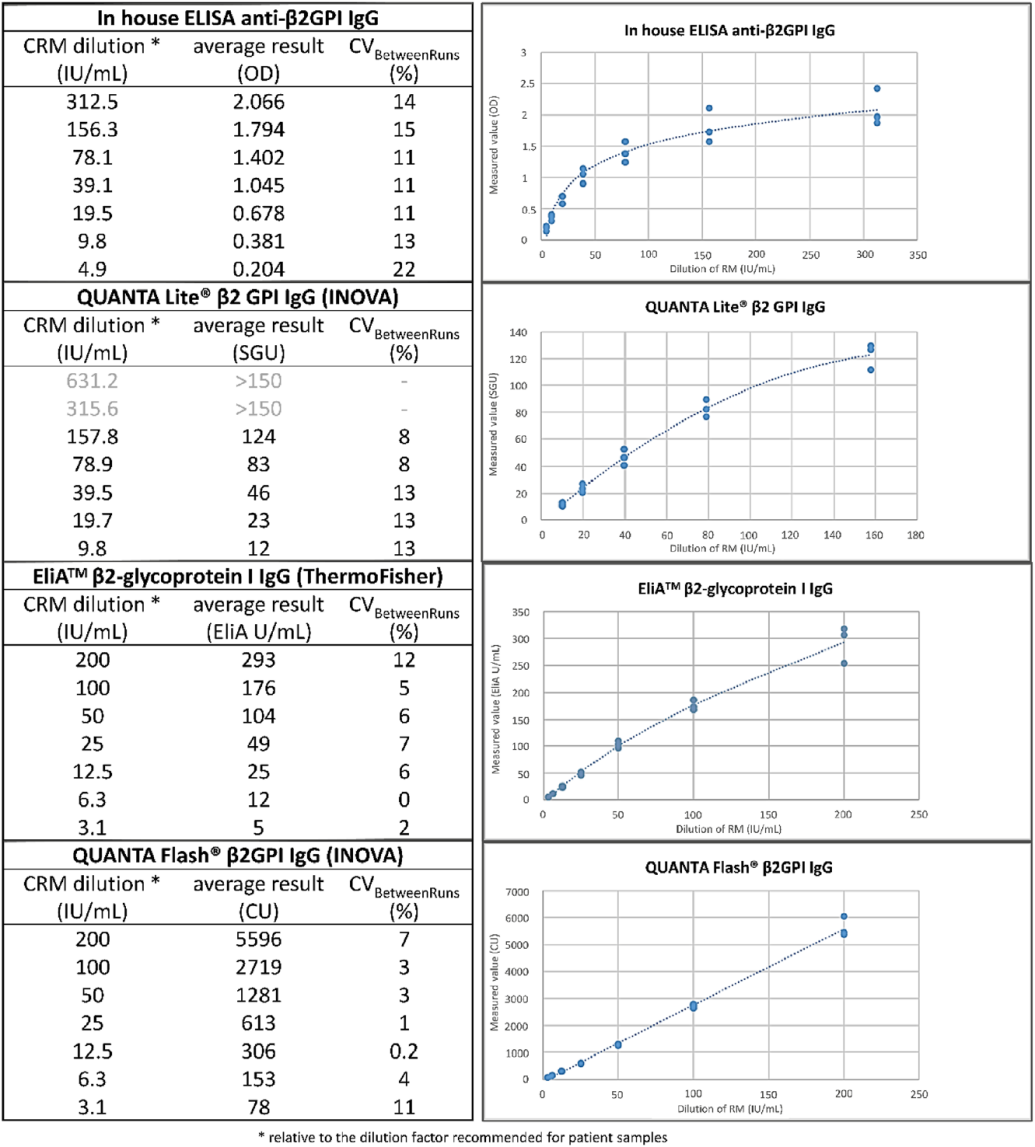
Mathematical relationship between assay-specific units and CRM’s IU/mL for 4 assays measuring anti-β2GPI IgG. According to the manufacturer’s specifications 200 IU/mL is the value attributed to the CRM tested at the specific-assay recommended dilution factor for patient samples (100 % dilution condition), respectively 1:50 for in-house β2GPI ELISA, 1:101 for QUANTA Lite^®^ β2GPI, and undiluted (not taking the applied instrument dilution factor into account) for EliA™ β2-Glycoprotein I and QUANTA Flash^®^ β2GPI. In agreement with this, we tested the CRM at the following IU/mL concentrations (corresponding to the specific dilution applied) in each of the four assays: in-house β2GPI IgG ELISA: 312.5 IU/mL (1:32), 156.3 IU/mL (1:64), 78.1 IU/mL (1:128), 39.1 IU/mL (1:256), 19.5 IU/mL (1:512), 9.8 IU/mL (1:1,024), 4.9 IU/mL (1:2048). QUANTA Lite^®^ β2GPI IgG: 631.2 IU/mL (1:32), 315.6 IU/mL (1:64), 157.8 IU/mL (1:128), 78.9 IU/mL (1:256), 39.5 IU/mL (1:512), 19.7 IU/mL (1:1,024), 9.8 IU/mL (1:2048). EliA™ β2-Glycoprotein I IgG and QUANTA Flash^®^ β2GPI IgG: 200 IU/mL (undiluted), 100 IU/mL (1:2), 50 IU/mL (1:4), 25 IU/mL (1:8), 12.5 IU/mL (1:16), 6.3 IU/mL (1:32), 3.1 IU/mL (1:64), corresponding dilution excludes the applied instrument dilution factor.

We included four assays providing quantitative or semi-quantitative measurement results for the concentration of anti-β2GPI IgG autoantibodies in human serum (see Table 2). One assay was an in-house developed ELISA [31] (details are reported in the supplementary file), while the other three were commercially available assays with a CE label for IVD use: specifically, QUANTA Lite^®^ β2GPI IgG (708665) and QUANTA Flash^®^ β2GPI IgG (701248) (Inova Diagnostics, San Diego, CA, USA), and EliA™ β2-Glycoprotein I IgG (Thermo Fisher Scientific, Freiburg, Germany). The commercial assays were performed according to the manufacturer’s instructions for use (IFU). The clinical samples were assayed at the recommended dilution, while the reconstituted CRM was assayed at seven different dilutions to generate a standard curve to convert the assay-specific units to the IU/mL units assigned to the CRM. These seven dilutions were measured in duplicate for each assay on three independent runs. Details are reported in Figure 3 legend.

**Table 2:** The four assays measuring the concentration of anti-β2GPI IgG autoantibodies in human serum included in the commutability assessment.

Assay name (product code)	Manufacturer/developer	Technology	Recommended dilution factor	Assay specific units	Measurement range	Cut-off value
In-house β2GPI ELISA	IAI-BS labs	Manual ELISA	1/50	OD values	0–2.5 OD	0.170 OD
QUANTA Lite^®^ β2GPI IgG (708665)	Inova diagnostics	Manual ELISA	1/101	SGU	0-150 SGU	20 SGU
EliA™ β2-Glycoprotein I IgG	Phadia, Thermo Fisher scientific	Automated FEIA	1/10 instrument dilution	EliA U/mL	0.8 to 480 EliA U/mL	10 EliA U/mL (7–9.9 equivocal)
QUANTA Flash^®^ β2GPI IgG (701248)	Inova diagnostics	Automated CLIA	1/11.9 instrument dilution	CU	6.4–6100 CU	20 CU

ELISA, enzyme-linked immunosorbent assay; FEIA, fluorescent enzyme immunoassay; CLIA, chemiluminescence immunoassay.

### Data analysis

The data analysis was performed using Microsoft Excel, its add-in software Analyse-it, and the GraphPad Prism 10 software (GraphPad Software, Boston, MA, USA).

For commercial assays, the assay-specific values for the CS and the CRM dilutions were calculated based on the assay–specific calibrators used, following the manufacturer’s instructions. The cut-off values applied to classify results as positive were also based on the values described in the IFUs.

For the in-house ELISA protocols, inter-run CV <15 % of specific medium/high positive controls and CV <20 % of low/borderline positive controls were used to accept the run [32]. The cut-off values were calculated by the 99th percentile method in 100 healthy donor serum samples.

For the conversion of the assay-specific units to IU/mL, a standard curve was generated for each of the four assays based on the results obtained for the seven dilutions of the CRM measured on three different runs. CRM dilutions that gave results above the measurement range of the specific assays were excluded as well as if one replicate measurement was an obvious outlier (suggesting a technical error). The standard curves were calculated using a 4-parameter sigmoidal fit using the GraphPad Prism 10 software. These standard curves were used to convert the results obtained for the 133 aPL positive samples and the 99 control samples into IU/mL units.

To further evaluate the commutability of the CRM with the aPL positive samples, the calibration effectiveness was evaluated as described in the IFCC Working Group Recommendations for assessing commutability [33]. The approach measures the variability between the various assays as the inter-measurement procedure bias range (IMPBR). It was not meaningful to calculate the IMPBR before the conversion to IU/mL as all assays have different units. After the conversion into IU/mL, the IMPBR was calculated as described below. From the 133 aPL positive samples, 78 were excluded from the analysis because no reliable numerical IU/mL results could be obtained for at least one of the assays. Nineteen of the excluded samples had measurement results that were above the measurement range (usually for all four assays) and for 59 samples the obtained measurement results were below the measurement range (usually for the in-house and QUANTA Lite^®^ ELISA, and EliA™ FEIA). The results of 55 samples were finally evaluated. For each CS, the trimmed mean value (excluding the highest and the lowest of the four assay results) was set as the target value. Then the relative difference (% bias) from the target was calculated for each of the 4 assay results obtained for that specific CS. For each assay, the median relative difference to the target value was calculated over the 55 CS samples. Finally, the IMPBR was calculated as the range between the assay with the highest and the lowest median relative difference to the target value.

In addition, we also evaluated whether conversion to the IU/mL could improve the agreement between the four assays by calculating new cut-off values. The results of the 99 control samples were used to establish the cut-off for the positive outcome following the recommendations of the International Committee in Sydney [8], 34].

## Results

### Characterization of CRM aPL profile

#### CRM’s anti-D1 and anti-D4-5 activity

CRM was tested against D1 undiluted (100 % percent dilution relative to the recommended assay dilution factor) and at two different dilutions, 1:4 (25 %), and 1:16 (6.3 %), not considering the applied instrument dilution factor. We obtained the following values: >1,380.4 CU (100 %), 426.3 ± 25.2 CU (25 %), and 124.5 CU (6.3 %). All three values were significantly greater than the cutoff of 19.9 CU. In contrast, CRM was negative/borderline for anti-D4-5 at the recommended dilution factor to test patient samples in the ELISA test, 1:101 (100 % percent dilution). Increasing the concentration of CRM to 157.8 % percent dilution (1:64 dilution) and 315.6 % (1:32 dilution) led to an apparent dose-dependent reactivity, yet such reactivity was weak and could not be confidently assigned to specific reactivity due to potential artifacts at high concentrations of serum. Thus, these results show significant CRM’s reactivity toward D1 and negligible reactivity towards D4-5. We report the data in the supplementary file (Supplementary Figure S2).

#### CRM’s activity against specific domain deleted β2GPI molecules

To confirm and expand the initial findings obtained with the fragments, CRM was tested using intact β2GPI purified from human serum, intact recombinant β2GPI, and five novel β2GPI domain-deleted variants. The monoclonal MBB2 antibody that targets D1 was used as a control. Unlike β2GPI purified from serum, recombinant β2GPIs used in this study carry an engineered tag at the N-terminus.

As expected from recent publications [27], 28], 30], MBB2 reacted equally well with β2GPI purified from serum, intact recombinant β2GPI, and four variants containing D1. However, it did not react against r-ΔD1, which lacks D1. This validates the reactivity of MBB2 and the functional integrity of the variants.

Similar to MBB2, CRM reacted well with β2GPI purified from plasma and intact recombinant β2GPI. However, unlike MBB2, the reactivity toward recombinant β2GPI was ∼20–40 % lower compared to β2GPI purified from serum, indicating that the engineered tag can interfere, either directly or indirectly by changing antigen orientation, with antibody binding. Most importantly, this suggests that anti-D1 autoantibodies are abundant in the CRM sample. In line with this hypothesis, the most significant loss of CRM reactivity was observed with r-ΔDI, followed by r-ΔDV and r-ΔDII. Notably, the same trend was measured for three full-blown APS serum samples. This indicates that CRM correctly captures the complex serological properties of these patients. We report these results in Figure 1.

#### CRM’s anti-cardiolipin activity

All three anti-CL assays detected high anti-CL IgG activity in CRM. Figure 2 shows QUANTA Flash^®^ CLIA, QUANTA Lite^®^ ELISA, and EliA™ CRM’s anti-CL IgG regression models. QUANTA Flash^®^ showed the best curve r-squared (R^2^=0.9968). QUANTA Lite^®^ and EliA™ reached lower r-squared values (respectively, R^2^=0.9572 and 0.9591). The two highest CRM concentrations were out of range for EliA cardiolipin IgG.

### Evaluation of the calibration effectiveness of the CRM

#### Results obtained for the CSs and the CRM applying the assay-specific calibration

Table 3 summarizes the qualitative results obtained for the 133 aPL positive and 99 control samples according to the assays-specific calibration and recommended cut-off values. These results show disagreement among the outcomes of the four assays in about 36 % of the aPL-positive samples. The QUANTA Flash^®^ β2GPI IgG gave the highest percentage of β2GPI IgG positive samples but also had a false positive rate of 7 % with mainly positive results in the serum samples of aPL negative SLE patients.

**Table 3:** Qualitative results obtained for the 232 CSs using the assay-specific calibration and cut-off values.

Diagnosis	Positive results						
	In-house ELISA (≥0.170 OD)	QUANTA Lite^®^ (≥20 SGU)	EliA™ β2GPI (≥10 EliA U/mL)	QUANTA Flash^®^ β2GPI (≥20.0 RLU)	All 4 assays	Discordant results	Accordant negative results
**aPL positive samples (n=133)**	**77 (58 %)**	**62 (47 %)**	**62 (47 %)**	**100 (75 %)**	**57 (43 %)**	**48 (36 %)**	**28 (21 %)**

aPL without SARDs (n=16)	6	5	6	10	4	7	5
aPL with SARDs (n=18)	8	6	7	12	6	6	6
PAPS with obstetrical (n=13)	7	7	7	11	6	6	1
PAPS with thrombotic (n=39)	28	26	25	34	24	11	4
PAPS with thrombotic and obstetrical (n=12)	9	7	7 (+1 equivocal)	10	6	5	1
SAPS with obstetrical (n=1)	0	0	0	1	0	1	0
SAPS with thrombotic (n=28)	14	6	8 (+1 equivocal)	16	6	11	11
SAPS with thrombotic and obstetrical (n=6)	5	5	5	6	5	1	0

**Control samples (n=99)**	**0**	**1 (1 %)**	**0**	**7 (7 %)**	**0 (0 %)**	**8 (8 %)**	**91 (92 %)**

aPL-negative SLE (n=24)	0	1	0	6	0	7	17
Healthy donors (n=75)	0	0	0	1	0	1	74

aPL, anti-phospholipid antibodies; PAPS, primary anti-phospholipid syndrome; SAPS, secondary anti-phospholipid syndrome; SARDs, systemic autoimmune rheumatic diseases; SLE, systemic lupus erythematosus.

#### CRM evaluation in anti-β2GPI IgG methods

For each of the four assays, seven dilutions of the CRM were measured on three different runs, and only the results within the measurement range of the method were retained. The relationship between the IU/mL concentrations and each specific method unit is shown in Figure 3. As expected, there was no good linear relation with the measurement results of the in-house assay in the high range, as reported units are OD values above 1.0. Also, the linear relation was not optimal for the QUANTA Lite^®^ and EliA™ β2GPI IgG (Pearson’s r=0.973 and 0.977, respectively). In contrast, there was a good linear relationship for the QUANTA Flash^®^ β2GPI IgG (Pearson’s r=0.993). To treat all the data the same, a 4-parameter sigmoidal fit was used for all assays to calculate the standard curve for converting the measurement results to the IU/mL.

**Figure 4:**
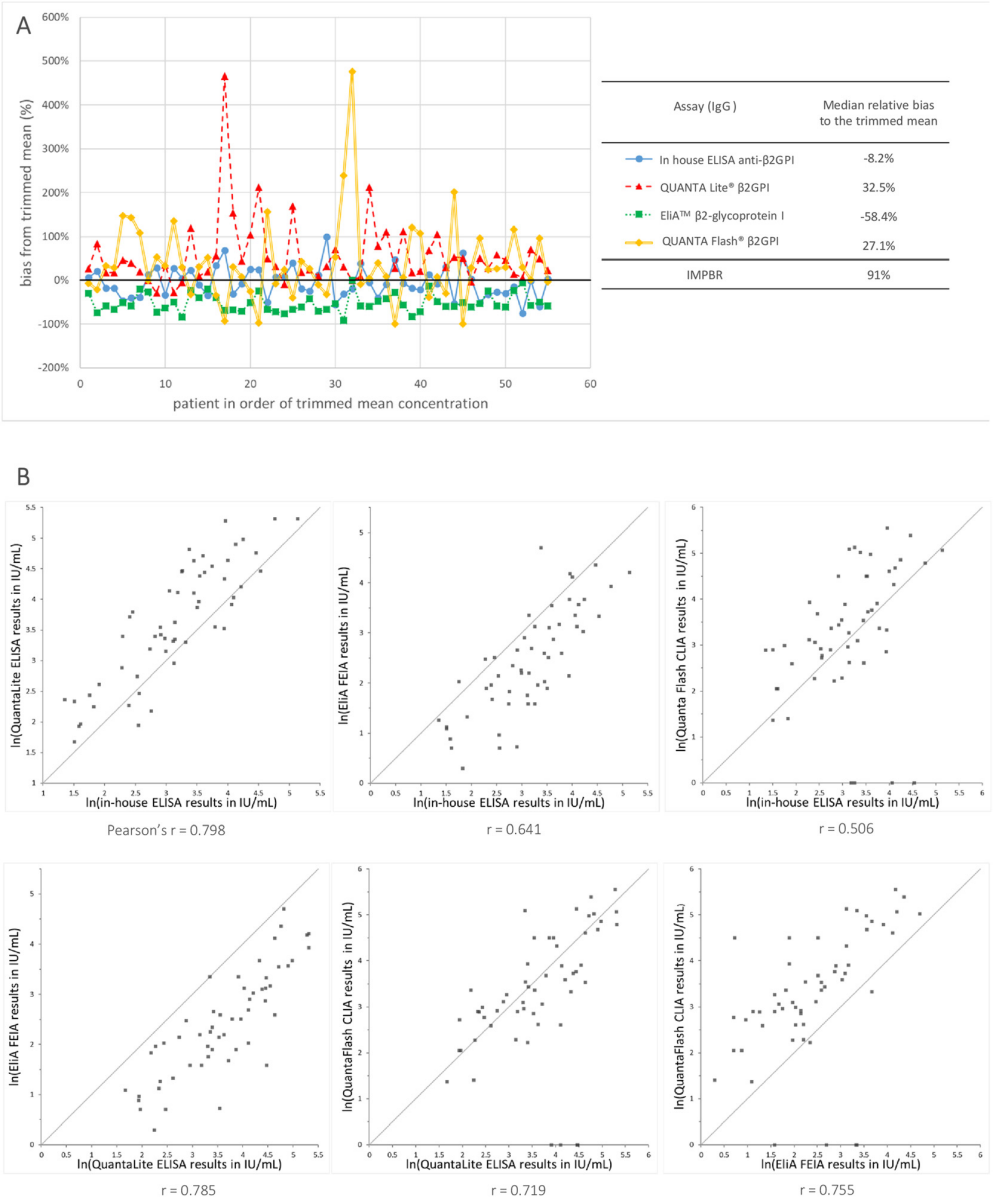
Agreement among the results obtained with 4 assays measuring anti-β2GPI IgG after mathematical recalibration to the IU/mL as defined by the CRM. (A) Difference in percent from the target value (trimmed mean) for the IU converted measurements results obtained with the 4 MPs measuring anti-β2GPI IgG in 55 aPL positive samples. (B) Scatter plots for each pair-wise assay comparison after the recalibration to the international units (IU/mL) as defined by the CRM. The scatter plots show the ln transformed measurement results and the grey line indicates the identity lines. Below each graph, the Pearson correlation coefficient (r) among the measurement results of both methods is shown.

The agreement among the quantitative measurement results of the four assays after the conversion to the IU/mL was evaluated based on the calculation of the IMPBR as described in IFCC Working Group recommendations for assessing commutability part 3 [35]. The results are shown in Figure 4. The EliA assay tended to give the lowest results, while the QUANTA Flash and the QUANTA Lite assays gave the highest results. The difference between the relative bias to the trimmed mean of the QUANTA Lite assay and the QUANTA Flash assay was only 5.4 %, suggesting that for these two assays, the conversion to the IU/mL has led to a good harmonization of the average results obtained for the 55 CSs in this study. The scatter plots shown in Figure 4 also confirmed this, and they also clearly indicated that (as expected) the conversion to IU cannot solve the issue of the scatter of the measurement results of the individual CSs. The difference between the relative bias to the trimmed mean of the other assays comparisons was larger, ranging from 35.3 to 91 %. The largest difference was observed between the EliA and the QUANTA Lite assay.

We also evaluated if the conversion to the IU/ml units would allow the use of a consistent cut-off value for all four assays and if this consistent cut-off value would improve the agreement among the qualitative outcomes of the assays. For each of the 4 assays, a new cut-off in IU/mL was calculated as the 99 % percentile of the measurement results obtained for the 99 control samples. The obtained cut-off values for the In-house ELISA, the EliA™ β2GPI IgG, and the QUANTA Flash^®^ β2GPI IgG were very close together: 3.05 IU/mL, 2.58 IU/mL, and 2.01 IU/mL, while QUANTA Lite^®^ β2GPI IgG had a much higher estimated cut-off:15 IU/mL.

When we applied the cut-off value of 3.0 IU/mL to the measurements results obtained for the in-house ELISA, the EliA, and the QUANTA Flash methods, we see that there is an improved concordance of the qualitative results for the aPL positive samples: 85 % (50 % positive and 35 % negative) in comparison with 67 % (46 % positive and 21 % negative) before (Table 4). The cut-off value of 3.0 IU/mL did not have a negative impact on the false positive rate of the three assays. However, the cut-off of 3.0 IU/mL was not suitable for the QUANTA Lite as it could lead to a false positive rate as high as 24 %, and false positive results were observed in both the SLE control samples and the healthy control samples.

**Table 4:** Improvement of the agreement among the qualitative measurements results.

Original assay-specific cut-off	Positive results (IgG)				Agreement among 3 assays (IgG): In-house ELISA, EliA, and QUANTA Flash^a^		
	In-house ELISA β2GPI	EliA™ β2GPI	QUANTA Flash^®^ β2GPI	QUANTA Lite^®^ β2GPI	Accordant positive	Discordant results	Accordant negative
aPL positive samples (n=133)	77 (58 %)	62 (47 %)	100 (75 %)	62 (47 %)	61 (46 %)	44 (33 %)	28 (21 %)
Control samples (n=99)	0	0	5 (5 %)	1 (1 %)	0 (0 %)	8 (8 %)	94 (95 %)

**Common cut-off (3 IU/mL)**	**Positive results (IgG)**				**Agreement among 3 assays (IgG): In-house ELISA, EliA and QUANTA flash**		

	**In-house ELISA** **β2GPI**	**EliA™** **β2GPI**	**QUANTA Flash** ^**®**^ **β2GPI**	**QUANTA Lite** ^**®**^ **β2GPI**	**Accordant positive**	**Discordant results**	**Accordant negative**

aPL positive samples (n=133)	79 (59 %)	71 (53 %)	77 (58 %)	90 (68 %)	66 (50 %)	20 (15 %)	47 (35 %)
Control samples (n=99)	2 (2 %)	0 (0 %)	1 (1 %)	24 (24 %)	0 (0 %)	3 (3 %)	97 (97 %)

^a^The results for the QUANTA Lite were not included as the threshold of 3 IU/mL seems not appropriated for this assay.

## Discussion

The heterogeneity of anti-β_2_GPI antibodies and the unclear nature of their epitopes present significant challenges in identifying standards that effectively reflect this variance. However, establishing such standards is crucial for the standardization and harmonization of laboratory tests used to diagnose APS, which continues to pose clinical difficulties. Notably, recent progress has led to the identification, in 2022, of a material represented by two identical reference preparations, NIBSC 21/266 and ERM^®^-DA477/IFCC, collectively referred to as CRM. This material has been validated to contain IgG antibodies to β2GPI and was produced in accordance with the requirements outlined in ISO_15194:2009 [36], 37], including commutability with CSs. Thus, CRM is positioned as a promising international calibrator for measuring anti-β2GPI IgG antibodies; however, further validation is required.

In our study, we advocate for the use of CRM as a tool for the standardization and harmonization of anti-β2GPI IgG antibody tests. Our independent studies confirm that CRM contains polyclonal anti-β2GPI IgG antibodies. Moreover, our findings demonstrate that these antibodies recognize β2GPI in a manner that appears to be consistent with what we observed in three APS patients often encountered in clinical practice. Specifically, CRM and the APS serum samples show D1-dependent, followed by D5- and D2- dependent, anti-β2GPI IgG antibody reactivity. However, the nature of the epitopes at the basis of this, whether conformational or linear, as well as the affinity of these antibodies for β2GPI, remains to be elucidated and requires additional investigations. Also, whether these considerations can be generalized to a larger APS population remains to be established.

Overall, we can state that CRM show the typical strong anti-D1 reactivity of APS full-blown serum samples [11] confirmed by both the direct and the indirect experimental approaches of this work.

Moreover, CRM ERM^®^-DA477/IFCC displays strong reactivity in all the tested commercial anti-CL assays, supporting the value of CRM as a representative tool for β2GPI-dependent aPL occurring in patients.

This study also investigated if the common scale set by the CRM improves the comparability among four different assays measuring anti-β2GPI IgG. For two of the four assays, the mathematical recalibration of the measurement results to the IU/mL leads to a good comparability of the average result obtained for the CSs. It should be noted that these two assays have a different chemistry (ELISA vs. CLIA) but the same IVD company manufactures them. There could be similarities in the source and the preparation of the antigen, leading to a comparable epitope-specificity of the assays, which could make harmonization easier. However, even between these two assays the scatter of the measurement results of the individual CSs remains large. This suggests that the difference in selectivity is probably more complex and other factors like the solid phase, and the specific assay chemistry (pH, salt concentration, buffer composition in general) can have a role. For the other assay comparisons, there was a substantial relative bias between the average CS results expressed in IU/mL, and the largest difference was still about a factor 3 between the average CS results. The remaining bias can be caused by a difference in selectivity between the assays, but other factors might also play a role, as i) the use of the wrong calibration model (as we just applied a relatively simple mathematical recalibration model), ii) random error components like repeatability and between run imprecision.

Finally, we also evaluated the option of setting a common cut-off value expressed in IU/mL. For three of the four assays tested in this study, it is possible to set a common cut-off value, and this common threshold improves the accordance among the qualitative results of the assays. However, for one assay the cut-off was not suitable, as this would lead to a high rate of false positive results.

In conclusion, the results presented here show that ERM^®^-DA477/IFCC is representative of the heterogeneity anti-β2GPI/CL IgG antibodies present in the APS patients. This CRM can be a vital step in improving the agreement among the assays measuring anti-β2GPI IgG and reduces the very differences in dynamic ranges. On the other hand, a factor-3 and CS scatter variation among ELISA-based methods, CLIA and FEIA still remain after CRM dependent recalibration: it would be important to evaluate if these differences are clinically relevant. The recalibration of the assays to the International Units defined by CRM will not be sufficient to reach complete method harmonization when the assays have a different selectivity due to e.g. their variations in β2GPI epitope-domain exposure and their constituents. Additional research is needed to evaluate if the level of method harmonization that can be achieved with this CRM is sufficient to fulfill the clinical requirements for the anti-β2GPI IgG assays. If not, it will still be needed to use different cut-off values or reference intervals for the current assays and information of the assay used should be made available to the requesting clinician. In addition, the source of difference in selectivity of the assays should be further investigated as this is fundamental both in clinical practice and for the pushing forward of method harmonization.

## Supplementary Material

Supplementary Material
