# Supplementary material for: Antiphospholipid IgG Certified Reference Material ERM®-DA477/IFCC: a tool for aPL harmonization?
Source: Clin Chem Lab Med. 2025 Mar 21;63(7):1315–26. doi: 10.1515/cclm-2025-0032 (PMC12118607; doi:10.1515/cclm-2025-0032)
Supplement: Supplementary file 1 — Supplementary Material [file j_cclm-2025-0032_suppl_001.pdf]

Supplementary Material

Supplementary Methods

Anti-β2GPI and anti-D4-5 IgG detecting in-house ELISA protocol

We diluted β2GPI (in-house purified from a pool of 100 healthy donor serum samples, [1]) to a final concentration of 10 μg/ml or the D4-5 peptide (kindly produced and gifted by INOVA Diagnostics) to 2.5 μg/ml, in 0.05 M Calcium Carbonate pH 9.6.

We added 100 μl/well of the diluted antigen (β2GPI or D4-5) to a 96-multiwell plate (Thermo Fisher Scientific Cat.n° 95029180, Breakable Strip 1x8 EB) and incubated it overnight at 4°C.

The next day, we drained, blocked with 100 μl/well PBS/ 1% BSA/ 0.05% NaN3, and incubated the plate for one hour RT.

Meanwhile, we centrifuged thawed positive/negative controls, and patient serum samples 5600 RCF 10 min RT, to exclude potential particles/debris which could interfere with the test, and then diluted them at the recommended dilution factors of each assay: 1:50 for anti-β2GPI or 1:101 for anti-D4-5 IgG detection in PBS/0.05% Tween/0.05% NaN3 (sample diluent). We prepared water-reconstituted CRM in sample diluent at the following conditions:

- for anti-β2GPI IgG detection, at 312.5 IU/mL (1:32), 156.3 IU/mL (1:64), 78.1 IU/mL (1:128), 39.1 IU/mL (1:256), 19.5 IU/mL (1:512), 9.8 IU/mL (1:1024), 4.9 IU/mL (1:2048), According to the manufacturer’s specifications 200 IU/mL is the value attributed to CRM tested at the assay recommended dilution factor for patient samples
- for anti-D4-5 IgG detection, at 315.6 % (1.32), 157.8 % (1:64), 78.9 % (1.128), 39.4 % (1:256), and 19.7 % (1:512), percent dilution relative to assay recommended dilution factor

After blocking incubation, we drained the plate and added each diluted sample in duplicate, 100 μl/well. We left the plate for two hours at RT.

Near the end of sample incubation, we diluted the conjugate antibody anti-Human IgG (γ-chain specific) −Alkaline Phosphatase antibody produced in goat (Sigma-Aldrich) 1:1000 in PBS/0.05% Tween/0.05% NaN3.

We drained and washed the plate with PBS/0.05% Tween/0.05% NaN3, 150 µl/well three times, and added 100 µl/well of conjugate solution. We incubated the conjugate for one hour and thirty minutes at RT.

We drained and washed the plate three times with PBS/0.05% Tween/0.05% NaN3, 150 µl/well.

We prepared the substrate solution dissolving 1 Phosphatase Substrate tab (Sigma-Aldrich) per 5 ml 0.05 M Magnesium Carbonate pH 9.8. We drained the plate and added the substrate solution, 100 µl/well.

We incubated the plate at 37°C for 30,40 and 50 mins: at each incubation time, we performed a 405nm reading. The approximate OD cut-off value set at the 99th percentile, obtained in 100 healthy control subjects, is 0.170 OD for anti-β2GPI and 0.405 OD for anti-D4-5 IgG Detection.

CRM's activity against whole β2GPI and β2GPI variants lacking specific domains

We diluted in-house purified β2GPI, recombinant β2GPI wild-type (r-WT) and β2GPI variants lacking specific domains, in 0.05 M Calcium Carbonate pH 9.6 to a final concentration of 2.5 µg/ml and added them to 96 well ELISA plate (Thermo Fisher Scientific Cat.n° 95029180, Breakable Strip 1x8 EB) 100 µl/well. The plate was incubated overnight at 4°C.

The next day, we drained the plate and blocked it with 100 µl/well PBS/ 1% BSA/ 0.05% NaN3 for one hour RT.

Meanwhile, we diluted samples in PBS/0.05% Tween/0.05% NaN3 (sample diluent).

APS patient serum samples were centrifuged 5600 RCF 10 min RT and then diluted at the dilution factor recommended for this assay, 1:50. We diluted water-reconstituted CRM in sample diluent at

the following concentration percents, relative to the dilution factor recommended: 156 % and 39 %. We tested the monoclonal anti-D1 IgG antibody (MBB2) at the second-highest concentration (2.5 μg/ml) of the dose-dependent anti-β2GPI monoclonal reactivity reported in the literature [2]. After blocking incubation, we drained the plate and added the diluted samples in duplicate, 100 μl/well, incubating them for two hours RT.

Near the end of sample incubation, we diluted 1:1000 the conjugate antibody anti-Human IgG (γ-chain specific) −Alkaline Phosphatase antibody produced in goat (Sigma-Aldrich) in PBS/0.05% Tween/0.05% NaN3.

At the end of sample incubation, we drained the plate and washed it three times with PBS/0.05% Tween/0.05% NaN3, 150 μl/well. We added the conjugate solution 100 μl/well and incubated the plate for one hour and 30 min RT.

After conjugate incubation, we drained the plate and washed it three times with PBS/0.05% Tween/0.05% NaN3, 150 μl/well.

We dissolved one tab of phosphatase substrate (Sigma-Aldrich) per 5 ml 0.05 M Magnesium Carbonate pH 9.8 to prepare the substrate solution and added 100 μl/well to the drained plate.

The plate was incubated at 37°C for 30-90 minutes and read at 405nm after each incubation time reported.

Supplementary Figures

Figure S1: CRM preparation. Before testing, we equilibrated water reconstituted 4°C stored CRM at room temperature for at least 1 hour, and gently inverted the vial five times (according to ERM®-DA477/IFCC certificate). Subsequently, we diluted CRM in the sample diluent of the specific assay to prepare different concentrations of CRM. We tested each condition in duplicate on three independent runs. For instrumental methods, we independently diluted CRM, preparing 500 µl total volume per condition, and then we split each sample into two tubes (250 µl/tube) to test duplicates. We re-used residual water reconstituted CRM and residual CRM dilutions for the independent runs, storing samples at 4°C between two consecutive runs when necessary. For ELISA methods, due to the smaller quantity of CRM to add, we serially diluted CRM and freshly prepared each condition at every test.

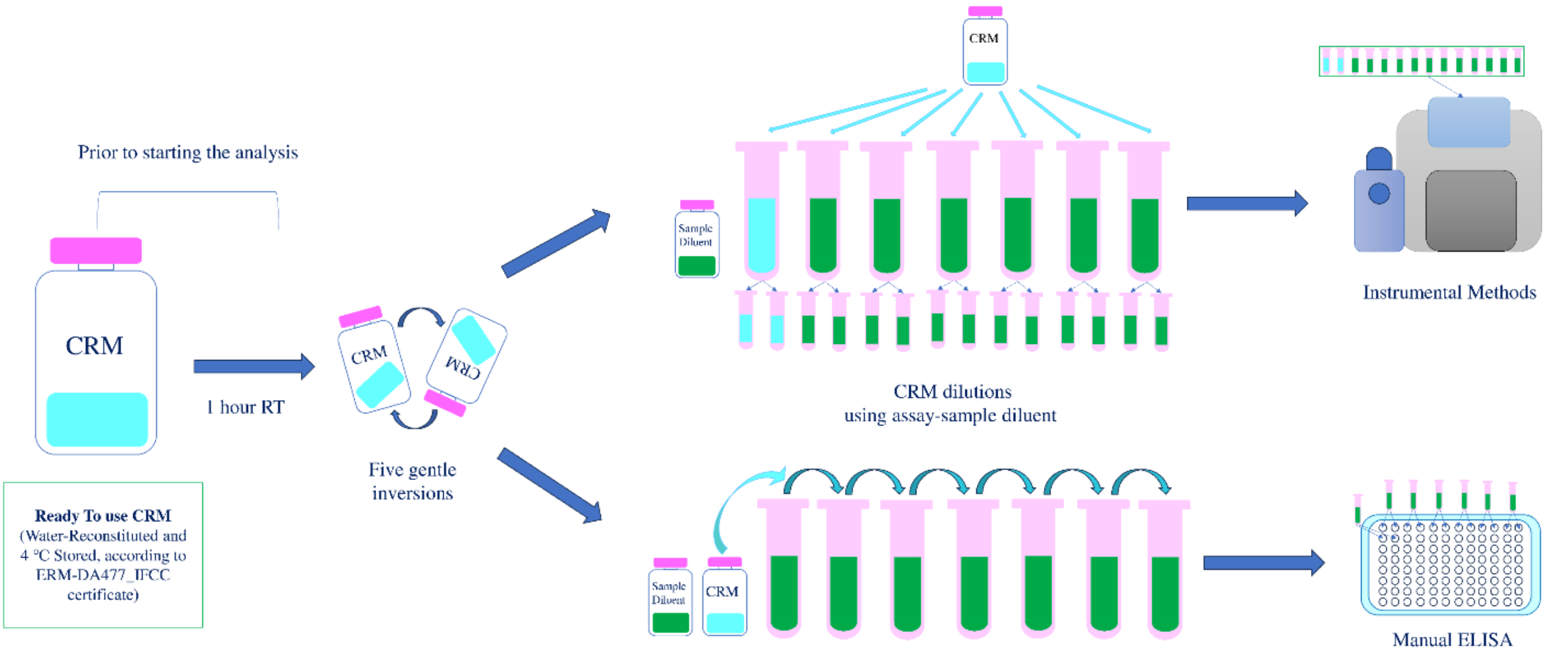

Figure S2: CRM's activity against D1 and D4-5 of β2GPI

A) For QUANTA Flash® β2GPI-Domain 1 (701188) assay, we diluted water-reconstituted CRM in sample diluent and tested it at 100 % (undiluted), 25 % (1:4), and 6.3 % (1:16) percent dilution, relative to the dilution factor recommended to test serum samples (undiluted = 100%). We repeated the 25% condition in three independent assays.

B) For in-house anti-β2GPI D4-5 IgG ELISA detection, we diluted water-reconstituted CRM in sample diluent at the following percent concentrations, relative to the dilution factor recommended to test serum samples (1:101 = 100%): 315.6 % (1:32), 157.8 % (1:64), 78.9 % (1:128), 39.4 % (1:256), and 19.7 % (1:512). We tested all the conditions in duplicate in three independent runs.

QUANTA Flash® anti-Domain1 IgG (INOVA)		
CRM dilution * (%)	average CU	$CV_{BetweenRuns}$ (%)
100	> 1380.4	-
25	426.3	6
6.3	124.5	-

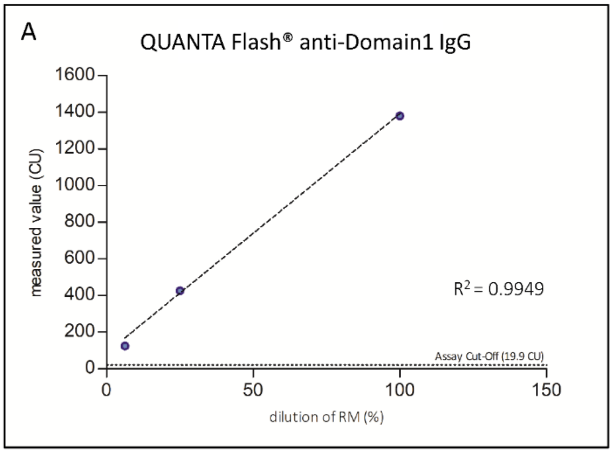

ELISA anti-Domain4-5 IgG (in-house)		
CRM dilution * (%)	average OD	$CV_{BetweenRuns}$ (%)
315.6	0.687	15
157.8	0.468	16
78.9	0.284	10
39.4	0.159	12
19.7	0.087	13

* relative to the dilution factor recommended for patient samples

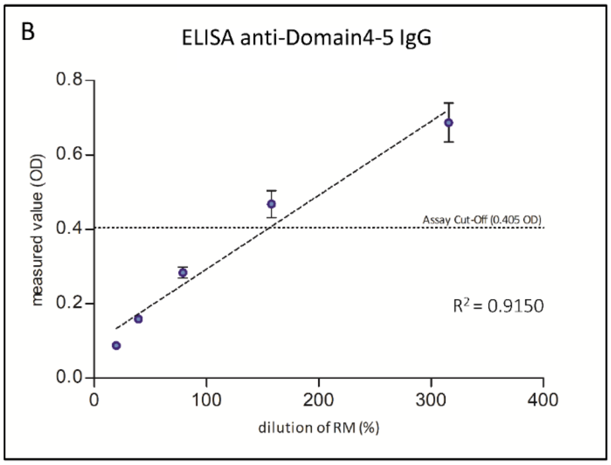

References

1. Artenjak A, Leonardi A, Križaj I, Ambrožič A, Sodin-Semrl S, Božič B, et al. Optimization of unnicked β2-glycoprotein I and high avidity anti-β2-glycoprotein I antibodies isolation. J Immunol Res. 2014;2014:195687.
2. Agostinis C, Durigutto P, Sblattero D, Borghi MO, Grossi C, Guida F, et al. A non-complement-fixing antibody to β2 glycoprotein I as a novel therapy for antiphospholipid syndrome. Blood. 2014;123(22):3478-87.
